# Lenalidomide in the Treatment of Lymphoproliferative Disorders and Multiple Myeloma

**DOI:** 10.1155/2013/812605

**Published:** 2013-04-16

**Authors:** Anna Marina Liberati, Umberto Vitolo, Antonio Palumbo, Agostino Cortelezzi

**Affiliations:** ^1^Medical Genetics Unit, University of Perugia, S. Andrea delle Fratte, Perugia, Italy; ^2^Dipartimento di Ematologia, Azienda Ospedaliero-Universitaria San Giovanni Battista di Torino, Torino, Italy; ^3^Fondazione IRCCS Ca' Granda Ospedale Maggiore Policlinico, Pad. Marcora, Via F. Sforza 35, 20122 Milano, Italy

Immunomodulating agents (ImiDs) are a novel class of anticancer drugs that have demonstrated impressive antitumor activity in various malignant disorders. Of this class, most recent research has been focused on the remarkably active agent, lenalidomide. Lenalidomide was designed to enhance immunologic and anticancer properties while potentially decreasing neurotoxic and teratogenic adverse effects of the parent compound thalidomide. The introduction of this novel agent has broadened the therapeutic landscape of hematologic malignant disorders including multiple myeloma (MM) and, more recently, other B-cell neoplasms. In this issue, we focused on mechanisms of action and results from clinical investigation that report the relevance of lenalidomide for the treatment of B-cell disorders including MM, chronic lymphocytic leukaemia (CLL), and non-Hodgkin's lymphomas by offering new mechanisms for targeting these diseases. In particular, papers defining the role of lenalidomide as part of the induction therapy before hematopoietic stem cells transplantation (HSCT) as well as its use as extended maintenance therapy post-HSTC in patients with MM will be of great interest. 

In this special issue, we invited papers on potential topics including, but are not limited to, lenalidomide: mechanism of action, lenalidomide as part of the induction therapy before HSCT and its use as consolidation and maintenance therapy of multiple myeloma, lenalidomide in chronic lymphocytic leukaemia, lenalidomide in diffuse large B-cell lymphoma, and lenalidomide in mantle cell and low-grade non-Hodgkin's lymphomas. Ten articles discuss the role of lenalidomide in the treatment of malignant disorders.

The paper entitled “*Lenalidomide in diffuse large B-cell lymphoma*” by C. Thieblemont et al. presents the biological rational for the use of lenalidomide in DLBCL in light of recent advances in the pathophysiology of the disease and the therapeutic results of the most recent trials published in literature or reported in meetings in relapsed/refractory situations as well as in first-line treatment. The paper entitled “*Practical approaches to the use of lenalidomide in multiple myeloma: a Canadian consensus*” by D. Reece et al. discuss the use of lenalidomide in the management of RRMM in the Canadian environment. The Chair (DR) invited panelists to research and write individual sections of the paper. The various sections were collected, compiled, and distributed to the group, which discussed the paper via web conference. Panelists subsequently generated a revised draft in which all sections included specific clinical guidance (i.e., practice considerations). The revised paper was discussed at a final web conference, where all practice recommendations were considered, revised as appropriate, and ultimately adopted by the full panel; any areas of disagreement are noted. Celgene Canada provided the impetus for the panel to pursue this project freely and independently. Celgene Canada supported the process throughout, including support for the participation of a medical writer (JA) in preparing this paper. The opinions represented here are solely those of the physician-panelists.

The paper entitled “*Biological activity of lenalidomide and its underlying therapeutic effects in multiple myeloma*” by R. Martiniani et al. is about the direct and indirect antitumor effects of lenalidomide on malignant plasmacells, bone marrow microenvironment, bone resorption, and host's immune response. The molecular mechanisms and targets of lenalidomide remain largely unknown, but recent evidence shows cereblon (CRBN) as a possible mediator of its therapeutical effects.

The paper entitled “*Molecular action of lenalidomide in lymphocytes and hematologic malignancies*” by J. M. McDaniel et al. summarizes the current information about lenalidomide in proliferative neoplasms and describes our understanding of the molecular mechanism of action in lymphocytes. Based on the overwhelming success of lenalidomide for the treatment of several hematologic malignancies, there is potential for therapies that augment host immune responses to be extended from the relapsed and refractory setting, to primary therapy.

The paper entitled “*Secondary primary malignancies in multiple myeloma: an old nemesis revisited*” by J. Yang et al. reviews the developmental history of myeloma therapy, with particular emphasis on the risk of secondary cancers, and examine the available data with regard to the risk of SPMs seen with lenalidomide. We also speculate about the mechanism(s) by which lenalidomide could increase the risk of second cancers. To conclude, we make some recommendations about how our current understanding affects our treatment decisions and suggest directions for future research. As new data emerge about lenalidomide and the risk of SPMs, it is our hope that this paper will help to put that information in proper perspective.

The paper entitled “*Lenalidomide in the treatment of young patients with multiple myeloma: from induction to consolidation/maintenance therapy*” by B. Lupo et al. presents an overview of the results achieved with lenalidomide-containing combinations in patients eligible for high-dose therapies, namely, young patients. The advantages obtained should always be outweighed with the toxicity profile associated with the regimen used. Therefore, here, we will also provide a description of the main adverse events associated with lenalidomide and its combination.

The paper entitled “*Lenalidomide in the treatment of chronic lymphocytic leukemia*” by A. Cortelezzi et al. provides a comprehensive summary regarding mechanism of action, efficacy, and safety of lenalidomide in CLL patients. Relevant clinical trials using lenalidomide alone or in combination are discussed. Lenalidomide shows good activity also in relapsed/refractory or treatment-naive CLL patients. Definitive data from ongoing studies are needed to validate overall and progression-free survival. The toxicity profile might limit lenalidomide use because it can result in serious side effects, but largely controlled by gradual dose escalation. Further understanding of the exact mechanism of action in CLL will allow more efficacious use of lenalidomide alone or in combination regimens.

The paper entitled “*Lenalidomide in diffuse large B-cell lymphomas*” by A. Chiappella et al. reports the most relevant clinical trials for the use of lenalidomide in DLBCL. Monotherapy with lenalidomide showed an activity in terms of overall response rate, with acceptable hematological and extrahematological toxicities in relapsed/refractory aggressive NHL. The role of lenalidomide as salvage therapy in both cell of origin patterns in DLBCL (germinal center B cell/activated B cell) was reported in preliminary data. Preliminary data regarding the role of lenalidomide in addition to chemoimmunotherapy (R-CHOP) in first-line clinical trials were discussed; data of safety, feasibility, and efficacy were promising.

The paper entitled “*Therapeutic activity of lenalidomide in mantle cell lymphoma and indolent non-Hodgkin's lymphomas*” by M. Gunnellini et al. discusses the role of lenalidomide in the therapeutic armamentarium of patients with indolent NHL or MCL.

The tenth paper entitled “*Lenalidomide before and after autologous hematopoietic stem cell transplantation in multiple myeloma*” by S. A. Tuchman et al. summarizes existing data that pertains to lenalidomide in the specific context of ASCT, and we share our thoughts on how our own group applies these data to approach this complex issue clinically.

*Anna Marina Liberati* *Umberto Vitolo* *Antonio Palumbo* *Agostino Cortelezzi*

